# Characterization of a conjugative IncA plasmid co-harbouring *bla*_NDM-5_ and *bla*_VIM-1_ in a KPC-producing *Klebsiella pneumoniae* clinical isolate K27

**DOI:** 10.3389/fmicb.2026.1959757

**Published:** 2026-10-05

**Authors:** Zhenzhen Liu, Dandan Dong, Nan Jia, Yifan Li, Youhan Gao, Yuanqi Zhu

**Affiliations:** 1 Department of Laboratory Medicine, The Affiliated Hospital of Qingdao University, Qingdao, China; 2 Department of Clinical Chemistry and Laboratory Medicine, Hietzing Hospital, Vienna Healthcare Group, Vienna, Austria

**Keywords:** *bla*_KPC-2_, *bla*_NDM-5_, *bla*_VIM-1_, *Klebsiella pneumoniae*, triple carbapenemase

## Abstract

**Objectives:**

Multiple-carbapenemase-producing *Klebsiella pneumoniae* has emerged as a critical threat to global health due to limited treatment options and high mortality. This study aimed to characterize the genomic features of a triple-carbapenemase-producing *K. pneumoniae* clinical isolate.

**Methods:**

A carbapenem-resistant *K. pneumoniae* (CRKP) strain K27 was collected from a patient in a tertiary hospital in January 2025. Antimicrobial susceptibility testing, carbapenemase detection, S1 nuclease pulsed-field gel electrophoresis (S1-PFGE), conjugation experiments, and plasmid fitness cost assays were performed. Whole-genome sequencing (WGS) of strain K27 was carried out on Illumina and PacBio platforms, bioinformatic analyses were performed using tools such as PlasmidFinder, ResFinder and ISfinder to characterize plasmid architecture, including replicon typing, resistance gene profiling, and mobile genetic element identification.

**Results:**

Strain K27 was an extensively drug-resistant ST11 *K. pneumoniae* producing three carbapenemases (NDM, KPC and VIM). Both S1-PFGE and WGS revealed that strain K27 carried four plasmids. The *bla*_KPC-2_ gene was located on an IncFII_pHN7A8_:IncR hybrid plasmid (assigned as pK27-KPC2) and carried by a truncated transposon Tn*6296*, while the *bla*_NDM-5_ and *bla*_VIM-1_ genes were co-harboured on a single IncA plasmid (assigned as pK27-NDM5/VIM1), where they were carried by a truncated transposon Tn*125* and integron In916, respectively. The two corresponding genetic elements were separated by IS*CR1*, and this arrangement was highly similar to that of plasmid p24A20009_B_NDM. Conjugation experiments demonstrated that the plasmid pK27-NDM5/VIM1 was transferable at a frequency of (4.5 ± 1.2) × 10^−2^, and the resulting transconjugant was designated as K27-C. Independent sample *t*-tests further revealed significant differences between the transconjugant K27-C and the *Escherichia coli* J53Azi^R^ groups, both in the area under the curve (*t* = 3.63, *p* = 0.002) and in the maximum optical density (*t* = 2.57, *p* = 0.02).

**Conclusion:**

The finding of two different class B metallo-carbapenemase genes co-harboured on a single conjugative IncA plasmid in a KPC-producing ST11 *K. pneumoniae* clinical isolate is alarming. This co-production of three distinct carbapenemases in a single strain could pose considerable challenges for the selection of clinical anti-infective therapies. Furthermore, the isolate may pose a high risk of subsequent clonal dissemination and/or horizontal gene transfer, necessitating ongoing epidemiological surveillance.

## 1 Introduction

The predominant mechanism of carbapenem resistance in Gram-negative bacteria is the production of carbapenemases (Suay-García and Pérez-Gracia, 2019). The main families of carbapenemases include: KPC (*Klebsiella pneumoniae* carbapenemase), which was first identified in a CRKP isolate in the USA and has now disseminated globally, representing the predominant carbapenemase mechanism in Gram-negative bacteria (Yigit et al., 2001; Li et al., 2024); VIM (Verona integron-encoded metallo-β-lactamase), which was initially discovered in *Pseudomonas aeruginosa* in Italy and continues to diversify with novel variants (Lauretti et al., 1999; Matsumura et al., 2017), and is increasingly detected in such as *K. pneumoniae* and *Escherichia coli* (Matsumura et al., 2017). NDM (New Delhi metallo-β-lactamase); which was originally identified in a *K. pneumoniae* isolate from a patient with a urinary tract infection in New Delhi, India, and is now globally distributed among *Enterobacteriales* including *E. coli*, *K. pneumoniae*, *Enterobacter cloacae*, *Citrobacter freundii* and other species (Yong et al., 2009; Zhao et al., 2023). IMP (imipenemase) and OXA (oxacillinase) are the predominant types of carbapenemases associated with *P. aeruginosa* and *Acinetobacter baumannii*, respectively (Li et al., 2024; Hammoudi Halat and Ayoub Moubareck, 2020).

Carbapenemase genes are typically carried singly, leading to resistance against last-resort carbapenem antibiotics, a major public health concern (Sisay et al., 2025). However, the co-harbouring of two carbapenemases in a single isolate, such as KPC + NDM, KPC + OXA-48, NDM + OXA-48, and NDM + VIM, is being reported with an increasing frequency and is associated with a significantly higher in-hospital mortality rate (Li et al., 2024, 2026). The co-harbouring of NDM and VIM in a single strain has been reported in several Gram-negative bacteria, including *K. pneumoniae*, *E. coli*, *Enterobacter asburiae*, *Moellerella wisconsensis*, and *P. aeruginosa,* with cases identified in Greece, Italy, India, and China (Miao et al., 2018; Ahmad et al., 2020; Mattioni Marchetti et al., 2024; Mattioni Marchetti et al., 2025; Tsolakidou et al., 2025; Subramanian, 2026). The co-harbouring of triple carbapenemases has also been documented. In Egypt, 8.6% (9/105) of CRKP isolates co-harboured KPC, NDM, and OXA-48 (Gandor et al., 2022), while co-harbouring of NDM, OXA-48, and VIM was found in 2 of 16 carbapenem-resistant *Enterobacteriales* (CRE) strains (El-Defrawy et al., 2026). In the Caribbean, the prevalence of co-harbouring of KPC, NDM, and VIM increased from 1.1% (2018) to 3.6% (2022) (Thomas et al., 2022). Conversely, a nationwide study in China reported that co-harbouring of KPC, NDM, and IMP occurred in only 0.1% (2/1,918) of carbapenemase-producing Gram-negative bacteria (Li et al., 2024). Despite its rarity, the co-harbouring of triple carbapenemases in a single isolate may pose a significant public health threat. To our knowledge, detailed characterization of the genetic structures underlying such co-harbouring strains remains limited.

In this study, we identified a CRKP isolate K27 co-harbouring *bla*_KPC-2_, *bla*_NDM-5_ and *bla*_VIM-1_ genes. We subsequently performed a series of experiments to confirm the co-harbouring of *bla*_NDM-5_ and *bla*_VIM-1_ on a single conjugative plasmid, characterized their genetic features, and evaluated the fitness cost associated with the plasmid. Our findings underscore the value of genomic surveillance in strengthening hospital infection prevention and control strategies.

## 2 Materials and methods

### 2.1 Bacterial strain isolation and identification, and antimicrobial susceptibility testing

*Klebsiella pneumoniae* strain K27 was isolated from a sputum specimen from an elderly male patient with severe pneumonia. Notably, this was the only bacterial strain isolated from the patient during his hospitalization. Sputum specimen was inoculated onto blood agar, chocolate agar, and MacConkey agar plates. Cultures were incubated at 37 °C in 5% CO₂ for 18–24 h. Identification and antimicrobial susceptibility testing were performed using the VITEK Compact 2 system (BioMérieux, France). The pure cultures were aliquoted into sterile Eppendorf tubes and stored at −80 °C for further analysis. Subsequently, the antimicrobial susceptibility of the strain to aztreonam/avibactam, ceftazidime/avibactam, polymyxinB, tigecycline, ertapenem, imipenem, and meropenem was determined using broth microdilution (BMD) in accordance with the Clinical and Laboratory Standards Institute (CLSI) document M07-A10 (CLSI, 2015). MICs of ampicillin, ampicillin/sulbactam, piperacillin/tazobactam, cefazolin,ceftriaxone, ceftazidime, cefepime, cefotetan, aztreonam, amikacin, tobramycin, gentamicin, ciprofloxacin, levofloxacin, and sulfamethoxazole/trimethoprim were tested by Vitek2 AST-GN13 card (bioMérieux, France). The breakpoints were interpreted according to Clinical and Laboratory Standards Institute (CLSI) guidelines M100, 36th edition, 2026 (CLSI, 2026). FDA MIC interpretative breakpoints were used for tigecycline.

### 2.2 Carbapenemase detection

The NG-Test® CARBA 5 assays (NG Biotech, France) for carbapenemase detection were conducted by following the manufacturer’s instructions. This multiplex immunochromatographic assay qualitatively detects five major carbapenemases (KPC, OXA-48-like, VIM, IMP, and NDM) on a single test strip. The result was interpreted as positive when both the control line (C) and the corresponding test line(s) showed red bands; negative when only the control line was visible; and invalid if the control line was absent (Tarlton et al., 2023).

### 2.3 Conjugation experiments

Conjugation assays were carried out with strain K27 as the donor and the sodium azide-resistant *E. coli* J53Azi^R^ as the recipient cells, and conducted as described previously (Zhao et al., 2022a). Overnight cultures of donor K27 and recipient J53 Aziᴿ were grown in LB broth at 37 °C with shaking, adjusted to an optical density at 600 nm (OD₆₀₀) of approximately 1.0, and mixed in equal volumes (0.5 mL each) in 4 mL of fresh LB. The mixture was incubated at 37 °C for 18 h without shaking. Controls consisted of donor-only and recipient-only cultures plated on the same selective medium. Following incubation, the mating mixture was serially diluted in sterile PBS and plated on selective Tryptic Soy Agar plates supplemented with sodium azide (100 mg/L) and meropenem (2 mg/L) to select for transconjugants. Transconjugants (T) were counted as colonies on agar containing both antibiotics, while recipients (R) were those on agar with sodium azide alone. Conjugation assays were performed in ‌three independent biological replicates on separate days‌ to ensure reproducibility. Putative transconjugants were confirmed by ‌PCR, S1-PFGE, and NG-Test® CARBA 5‌. The conjugation frequency was calculated as ‌T/R‌. Carbapenemase gene detection was performed by PCR using the FilmArray‌ systemand its supporting reagent kit (bioMérieux, France).

### 2.4 S1-nuclease-pulsed-field gel electrophoresis (S1-PFGE)

The plasmids in both strain K27 and the transconjugants were detected by S1-PFGE, which were conducted as described previously (Zhao et al., 2022b). PFGE plugs were prepared in 1% SeaKem Gold agarose, digested with S1 nuclease (Takara, China), and run on a CHEF-Mapper XA system (Bio-Rad, USA) at 6 V/cm, 120° angle, 6.5–34.5 s switch time, 14 °C in 0.5 × TBE for 18.5 h. *Salmonella enterica* H9812 plugs digested with XbaI (Takara) served as size markers. Following electrophoresis, the agarose gel was stained with 4S GelRed nucleic acid stain (Sangon Biotech, China) and imaged using a Molecular Imager® Gel Doc™ XR+ gel documentation system (Bio-Rad).

### 2.5 Evaluation of plasmid fitness cost

Fitness cost of the plasmid was evaluated using growth curve, area under the curve (AUC) and maximum optical density (ODmax) as indicators (Alonso-del Valle et al., 2021; San Millan, 2018). All experimental procedures and result interpretation were performed according to previously described methods. A statistically significant reduction in at least one of the two parameters, AUC and ODmax, was defined as evidence of a fitness cost (Zhang et al., 2022). Growth curves for recipient *E. coli* J53Aziᴿ and transconjugants were determined in 96-well plates, each well containing 200 μL of bacterial suspension at 8 × 10^3^ CFU/mL. The plates were incubated at 37 °C in a SpectraMax ABS Plus (Molecular Devices, USA), with OD_600_ readings taken automatically every hour for 24 h. Sterile broth was used as the blank control. Statistical analyses were performed using SPSS version 23.0 on 9 independent biological replicates (25 time points per replicate), each with 3 technical replicates. Data normality and homogeneity of variance were verified using Shapiro–Wilk and Levene’s tests, respectively. Both assumptions were met (*p* > 0.05), justifying the use of parametric tests. An independent samples *t*-test was then used to compare the AUC and ODmax between transconjugants and *E. coli* J53Azi^R^. Graphs were generated using GraphPad Prism 10.

### 2.6 Whole-genome sequencing (WGS) and bioinformatic analysis

To obtain complete chromosomal and plasmid sequences of strain K27, we employed a hybrid sequencing approach combining Illumina and PacBio platforms (Xu et al., 2025). Genomic DNA was extracted using the Wizard Genomic DNA Purification Kit (Promega, USA). Integrity was verified by agarose gel electrophoresis, and concentration and purity were measured by NanoDrop 2000 spectrophotometer and Quantus Fluorometer.

#### 2.6.1 Library construction and genome sequencing

‌For Illumina sequencing, DNA was sheared to ~350 bp fragments using a Covaris M220 Focused Acoustic Shearer and libraries were prepared with the NEXTFLEX Rapid DNA-Seq Kit. The resulting libraries were subjected to paired-end sequencing (2 × 150 bp) on an Illumina NovaSeq 6000 platform (Illumina Inc., USA). For PacBio sequencing, DNA was fragmented to 8–10 kb using a PacBio Revio® instrument (Pacific Biosciences, USA) and the fragments were subsequently purified. Libraries were constructed using the SMRTbell Prep Kit 3.0 and sequenced on SMRT Cells according to the standard operating procedures available online.^1^

#### 2.6.2 Genome assembly

Raw Illumina paired-end reads were quality-filtered using fastp version 0.23.0 (Chen et al., 2018) with a 4-bp sliding window (Q20) and a minimum length cutoff of 50 bp. PacBio HiFi reads were combined with the clean Illumina reads for hybrid assembly using Unicycler version 0.4.8 (Wick et al., 2017), which resolves circularization automatically. The assembly was subsequently polished using Pilon version 1.22 (Walker et al., 2014) based on short-read alignments to correct base-level errors. Genome completeness and quality were evaluated using CheckM version 1.2.2 (Parks et al., 2015).

#### 2.6.3 Bioinformatic analyses

Species identification was confirmed by calculating average nucleotide identity (ANI) values via the EZBioCloud server (Yoon et al., 2017). Open reading frames (ORFs) and pseudogenes were predicted using RAST 2.0 (Aziz et al., 2008) and annotation was performed using BLASTn (Boratyn et al., 2013) and the NCBI Conserved Domains Database (CDD) (Wang J. et al., 2023). For BLASTn-based annotations, hits with nucleotide identity ≥95% and query coverage of 100% were retained (Coelho and Alves, 2022). For CDD searches, the default *E*-value threshold of 0.01 was applied (Wang J. et al., 2023). Antimicrobial resistance genes, mobile elements such as insertion sequences (IS), transposons, and integrons, and other features were annotated using ResFinder 3.2, PlasmidFinder 2.1, the Tn Number Registry, and the ISfinder online database (Dong et al., 2019). IS are short DNA segments containing at least one ORF that encodes a transposase, and are classified as either TIR-IS or non-TIR-IS depending on whether they are flanked by terminal inverted repeats (Zhou et al., 2008); unit transposons are usually larger than IS, flanked by IRs, and contain a transposase gene along with one or more internal “passenger” genes, which may encode antibiotic resistance (Partridge et al., 2018); Integrons are typically characterized by the presence of an integrase gene (intI) located near a primary recombination site known as attI (Bhat et al., 2025). Multilocus sequence typing (MLST) was carried out using the online database at https://bigsdb.pasteur.fr/. Known virulence factors and capsule loci were identified using Kleborate (v2.3.2) and the Kaptive command (Wyres et al., 2016; Lam et al., 2021). Gene organization was visualized using Inkscape 0.48.1.^2^

## 3 Results

### 3.1 Clinical features of the isolate

*Klebsiella pneumoniae* strain K27 was isolated in January 2025 from a sputum specimen of an 87-year-old male patient with severe pneumonia, who was hospitalised in a tertiary hospital in China. The patient had a one-week history of fever and pneumonia prior to admission but did not receive active treatment. He was admitted to the emergency department because of altered consciousness. After admission, piperacillin-tazobactam and linezolid were used as empirical therapy; however, the patient showed no clinical improvement. The patient eventually died of acute respiratory failure, which was subsequently complicated by systemic inflammatory response syndrome, septic shock and multiple organ dysfunction syndrome.

### 3.2 Antimicrobial susceptibility testing, carbapenemase detection, conjugation experiments and S1-PFGE

*Klebsiella pneumoniae* strain K27 was resistant to second-, third-, and fourth-generation cephalosporins, aztreonam, cephamycins, carbapenems, quinolones, and aminoglycosides, but remained susceptible to polymyxin B and tigecycline (Supplementary Table S1). The NG-Test® CARBA 5 assay showed that this strain was positive for three carbapenemases (NDM, KPC, and VIM; Supplementary Figure S1). The transconjugant (designated as K27-C), obtained by conjugation, was positive for two enzymes (VIM and NDM; Supplementary Figure S1) by NG-Test® CARBA 5 assay, and PCR further confirmed the presence of the corresponding *bla*_VIM_ and *bla*_NDM_ genes. S1-PFGE revealed that strain K27 carried four plasmids, whereas transconjugant K27-C carried only one plasmid of approximately 160 kb (Supplementary Figure S2). Additionally, the antimicrobial susceptibility test results showed that compared to *E. coli* J53Azi^R^, the transconjugant K27-C exhibited a higher increase in minimum inhibitory concentration (MICs) for ertapenem, imipenem, and meropenem (Supplementary Table S1). Furthermore, conjugation experiments showed that the strain K27 could transfer a plasmid harbouring the *bla*_VIM_ and *bla*_NDM_ genes (designated pK27-NDM5/VIM1) to *E. coli* J53 Azi^R^, with a transfer frequency of (4.5 ± 1.2) × 10^−2^.

### 3.3 Evaluation of plasmid fitness cost

Statistical analysis confirmed that both the AUC and ODmax data of the transconjugant K27-C group and the *E. coli* J53Azi^R^ group satisfied the assumptions of normality and homogeneity of variances (*p* > 0.05). Accordingly, an independent-samples t-test was performed. Results indicated statistically significant differences between the two groups for AUC (*n* = 9, *t* = 3.63, *p* = 0.002, Cohen’s *d* = 1.711, 95% CI: 0.597 to 2.788) and ODmax (*n* = 9, *t* = 2.57, *p* = 0.02, Cohen’s *d* = 1.214, 95% CI: 0.184 to 2.212). A comparative analysis of growth curves, AUC, and ODmax between transconjugant K27-C and *E. coli* J53Azi^R^ is presented in Figure 1.

**Figure 1:**
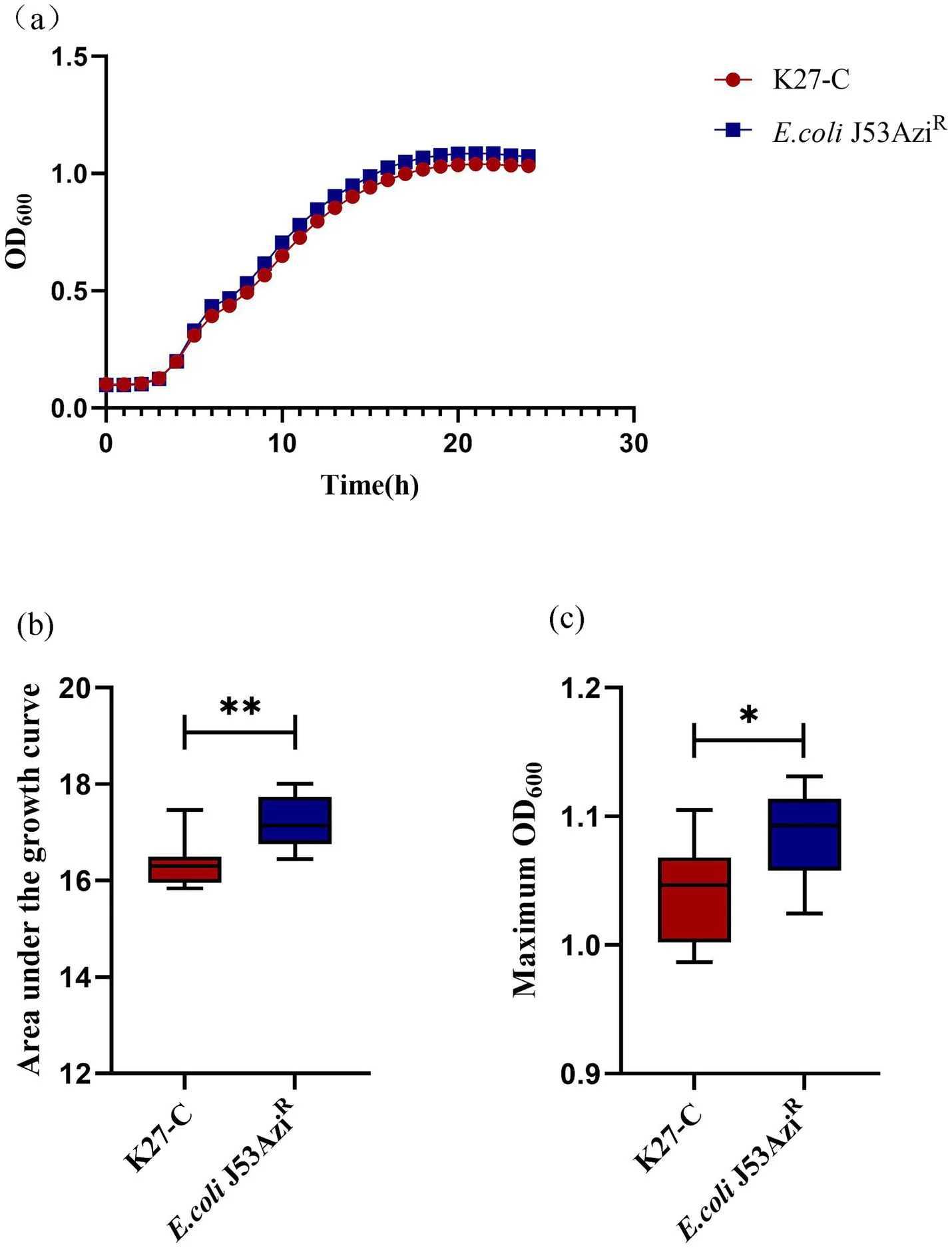
Comparison graph of conjugant K27-C and *Escherichia coli* J53Azi^R^ fitness cost. The growth curve **(a)**, the area under the curve **(b)**, and the maximum OD600 **(c)** of conjugant K27-C and *Escherichia coli* J53Azi^R^. Two-group comparison showed significant differences (*n* = 9 independent biological replicates; AUC: *t* = 3.63, *p* = 0.002; ODmax: *t* = 2.57, *p* = 0.02). Box plots show median, lower, and upper quartiles, the minimum value, and the maximum value. *p < 0.05; **p < 0.01.

### 3.4 General genomic characteristics of *Klebsiella pneumoniae* strain K27

The assembly quality of strain K27 meets the analysis requirements, as evaluated by metrics including coverage (100%) and N50 (11,952 bp). WGS of strain K27 revealed a 5,377,878 bp chromosome (designated as K27-chr) and four plasmids (designated as pK27-NDM5/VIM1, pK27-KPC2, pK27-3 and pK27-4, respectively). Comparing the chromosome sequence of strain K27 with the reference sequence of *K. pneumoniae* (GenBank GCA_000240185.2) yielded an ANI value of 99.85%, confirming that strain K27 is *K. pneumoniae*. Furthermore, strain K27 belonged to the ST11 (*gapA*_3, *infB*_3, *mdh*_1, *pgi*_1, *phoE*_1, *rpoB*_1, and *tonB*_4), and its chromosome harbours the β-lactamase gene *bla*_SHV-182_ and the fosfomycin resistance gene *fosA6*. Among the four plasmids carried by strain K27, two of them carry the genes for carbapenemase. According to its replication units, plasmid pK27-NDM5/VIM1 belongs to the IncA type plasmid, and it carries genes for resistance to β-lactams: *bla*_NDM-5_, *bla*_VIM-1_, *bla*_SHV-12_, and *bla*_TEM-1_; aminoglycosides: *aphA15*, *aadA1b*, *aacA4′* and *rmtB*; macrolides: *mph(A)*; phenicols: *catB2* and *floR*; sulphonamides: *sul1*; tetracyclines: *tetA*; and trimethoprim: *dfrA14b*. The second plasmid carrying the carbapenemase gene, pK27-KPC2, belonged to an IncFII_PHN7A8_:IncR hybrid plasmid harbouring genes for resistance to β-lactams: *bla*_KPC-2_, *bla*_SHV-12_, *bla*_TEM-1_, and *bla*_CTX-M-14_; and aminoglycosides: *rmtB*. The third plasmid, pK27-3 (84,840 bp), carried genes for resistance to β-lactams: *bla*_LAP-2_; sulphonamides: *sul1*; fluoroquinolones: *qnrS1*; tetracyclines: *tetA*; and trimethoprim: *dfrA14b*. The remaining plasmid, pK27-4 (197,201 bp), contained no known antimicrobial resistance genes (Table 1).

**Table 1:** Major features of chromosomes and plasmids analyzed.

Chromosome or plasmids	Total length (bp)	Mean G + C content (%)	Replicon type	Resistance genes
Chromosome				
pK27-chr	5,377,878	57.33		*bla*_SHV-182_, *fosA6*
Plasmids				
pK27-NDM5/VIM1	161,331	52.64	IncA	*bla*_**NDM-5**_, *bla*_**VIM-1**_, *bla*_SHV-12_, *bla*_TEM-1_, *aphA15*, *aadA1b*, *aacA4’*, *rmtB*, *mph(A)*, *catB2*, *floR*, *sul1*, *tetA*, and *dfrA14b*
pK27-KPC2	134,812	53.29	IncFII_pHN7A8_:IncR	*bla*_**KPC-2**_, *bla*_SHV-12_, *bla*_TEM-1_, *bla*_CTX-M-14_ and *rmtB*
pK27-3	84,840	54.11	IncHI1B	*bla*_LAP-2_, *sul1*, *qnrS1*, *tetA*, and *dfrA14b*
pK27-4	197,201	50.47	IncFII	No

### 3.5 Characteristics of plasmid pK27-NDM5/VIM1

pK27-NDM5/VIM1 is a circular plasmid (161,331 bp) containing 174 predicted ORFs (Figure 2, Table 1), with an average G + C content of 52.64%. Its replication initiation gene *repA* showed 98.32% nucleotide identity to that of pRA1 (GenBank accession no. FJ705807.1), confirming its assignment to the IncA family (Fricke et al., 2009). The plasmid backbone region (113,422 bp; excluding the accessory modules) shared 99.29% identity and 97% coverage with pRA1, but lacked an 11,564 bp region (corresponding to positions 15,968–27,531 bp of pRA1) containing plasmid maintenance genes. The accessory modules of 47,909 bp comprised a 46,714 bp multidrug-resistant (MDR) region and an IS*5* element (1,195 bp). The plasmid maintenance region of pK27-NDM5/VIM1 was disrupted by this IS*5*, whereas in pRA1 the plasmid maintenance and transfer regions are interrupted by Tn*6721* and IS*Ecp1*, respectively. Whole-plasmid comparison confirmed 73% query coverage and 99% nucleotide identity with pRA1, and the reduced whole-plasmid coverage was mainly attributable to the presence of accessory modules in pK27-NDM5/VIM1 that are absent or divergent in the reference plasmid pRA (Supplementary Figure S3).

**Figure 2:**
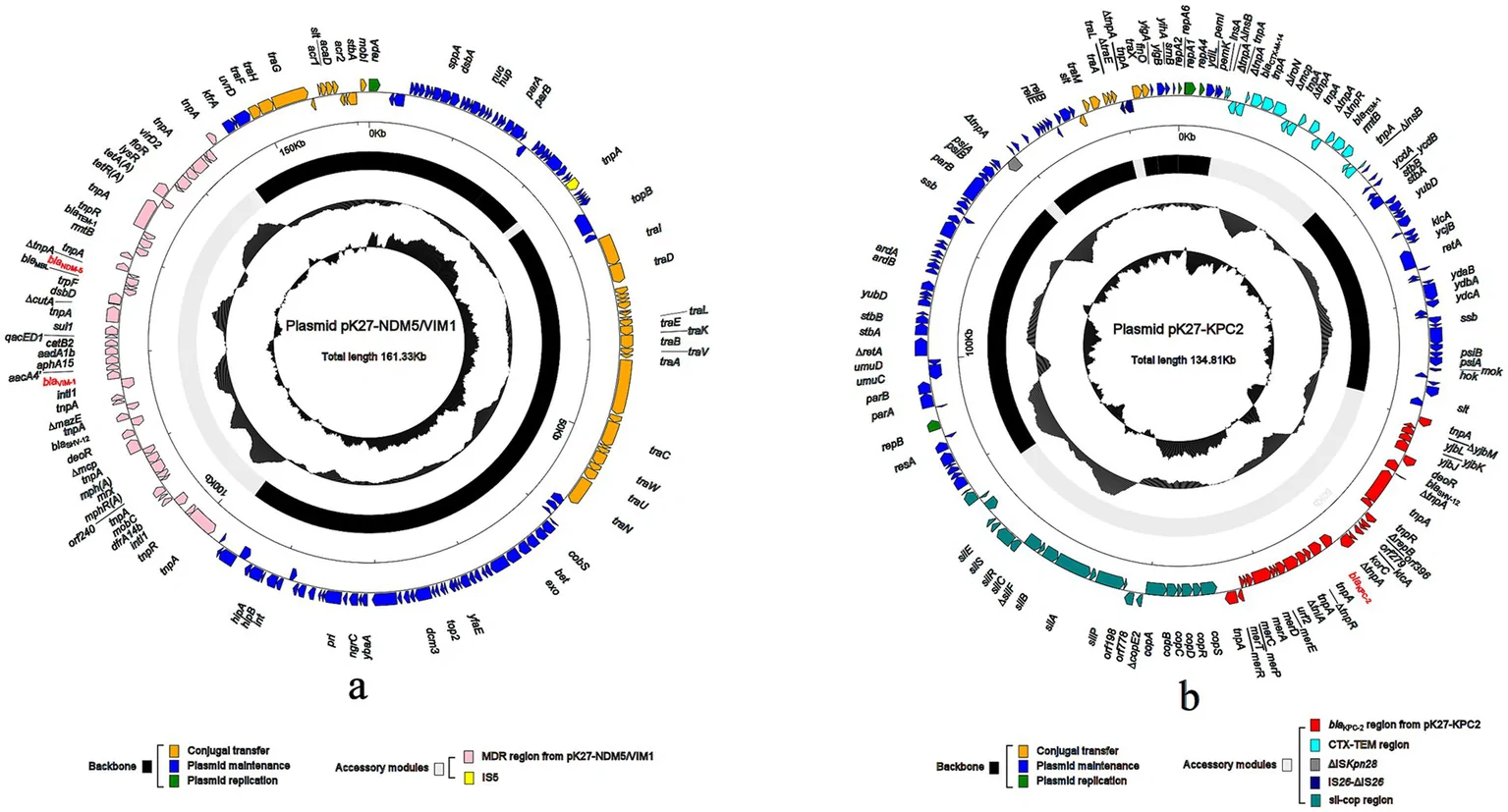
Schematic maps of plasmids pK27-NDM5/VIM1 and pK27-KPC2. **(a)** plasmid pK27-NDM5/VIM1; **(b)** plasmid pK27-KPC2. The plasmid map is oriented clockwise, with the origin (0 kb) positioned at the top (12 o’clock position). Genes are indicated by arrows. Different colors represent different backbone and accessory module regions. The innermost circle presents GC-skew [(G − C)/(G + C)], with a window size of 500 bp and a step size of 20-bp. The next-to-innermost circle shows GC content.

WGS analysis revealed that *bla*_NDM-5_ and *bla*_VIM-1_ were co-harboured within a 46,714 bp MDR region (positions 98,262–144,975 bp) on plasmid pK27-NDM5/VIM1. This MDR region was divided into three segments and further resolved into six modules based on the distribution of genetic elements and antimicrobial resistance genes. The three segments span positions 98,262–115,980; 115,981–129,072; and 129,073–144,975 bp, respectively. In order, the six modules comprise the *dfrA14b*-, *mph(A)*-, *bla*_VIM-1_/In916-, *bla*_NDM-5_/Tn*125*-, *rmtB*/*bla*_TEM-1_-, and *floR*/IS*CR2*-associated regions. (i) The *dfrA14b*-associated region derived from transposon Tn*1722*, where the *tnpR* and *mcp* genes were disrupted by the insertion of a ΔIn*191* element (carrying *dfrA14b*) and a truncated IS*26*-*mph(A)*-IS6100 unit. (ii) The *mph(A)*–associated region comprised three discrete structural elements: a truncated IS*26*-*mph(A)*-IS*6100* mosaic unit devoid of the 3′ inverted repeat terminal (IRT), a partially deleted IS*26*-*bla*_SHV-12_-IS*26* composite transposon remnant, and a truncated IS*Pa17* (ΔIS*Pa17*). (iii) The *bla*_VIM-1_/In916 region harboured *bla*_VIM-1_ along with the resistance genes *aacA4’*, *aphA15*, *aadA1b*, *catB2*, *qacEΔ1* and *sul1* on a truncated class 1 integron, ΔIn916. ΔISPa17 was located immediately upstream of ΔIn916, and IS*CR1* was positioned directly downstream. (iv) The *bla*_NDM-5_/Tn*125* region: *bla*_NDM-5_ resided within transposon ΔTn*125*, which was adjacent to IS*26* at its upstream end and to IS*CR1* at its downstream end, exhibiting the genetic organization IS*26*-ΔIS*Aba125*-*bla*_NDM-5_-*ble*_MBL_-*trpF*-*dsbD*-Δ*cutA*-IS*CR1*. (v) The *rmtB*/*bla*_TEM-1_ region comprised a Tn*2*-associated element harbouring the *rmtB* and *bla*_TEM-1_ genes, along with a ΔTn*1721* element carrying the tetracycline resistance gene *tetA*. (vi) The *floR*/IS*CR2* region contained an IS*CR2*-*floR* unit, a 2,927-bp remnant of Tn*6591* that shared a common IS*CR2* element with the downstream IS*CR2*-*floR* unit, and an IS*26* element (Figure 3, Supplementary Table S2).

**Figure 3:**
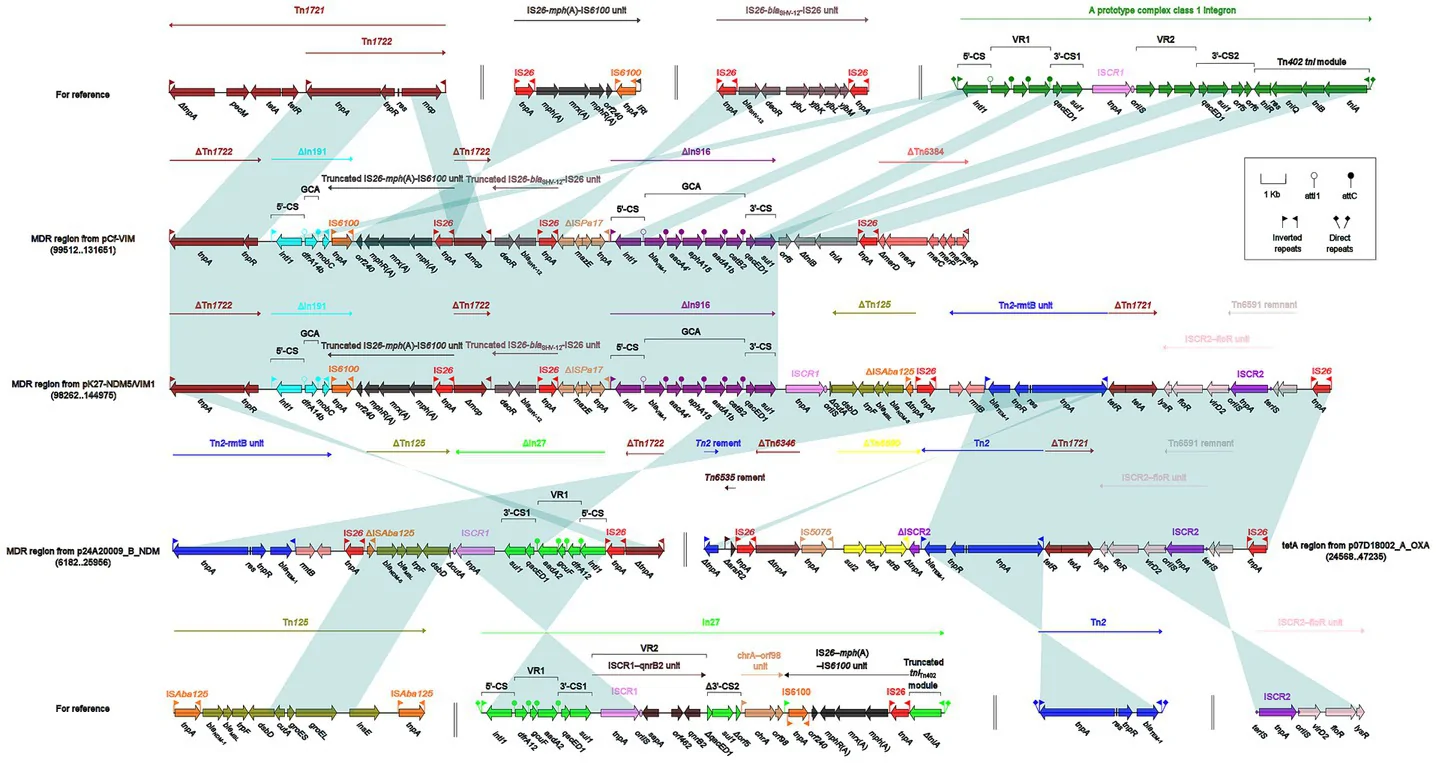
Comparison of the MDR regions of plasmids pK27-NDM5/VIM1 with those of the reference plasmids pCf-VIM, p24A20009_B_NDM, and p07D18002_A_OXA. Genes are denoted by arrows. Mobile elements, genes, and other features are colored based on function classification. Numbers in parentheses denote the nucleotide positions within the corresponding plasmids. Shading regions show nucleotide identity >95% calculated by BLASTn. The accession number of Tn*1721*, IS*26*-*mph(A)*-IS*6100* unit, IS*26*-*bla*_SHV-12_-IS*26* unit, Tn*125*, In27, Tn*2*, and IS*CR2*-*floR* unit for reference are X61367, KY270851, CP003684, JN872328, EF219134, HM749967 and CP042857, respectively.

To characterize the MDR region of plasmid pK27-NDM5/VIM1, a BLASTn analysis against the NCBI database was conducted. This analysis indicated that the MDR region did not align with any single plasmid sequence in its entirety. Instead, a segmented distribution was observed, with three distinct MDR segments matching different groups of plasmid sequences. (i) Fourteen sequences (Supplementary Table S3) from various species (including *C. freundii*, *Enterobacter hormaechei*, *Klebsiella oxytoca*, *E. coli*, *K. pneumoniae*, *Morganella morganii*, *Aeromonas* sp., and *Leclercia adecarboxylata*) showed high similarity (coverage: 56%; identity: >99%) within the 1–24,395 bp subregion. Consequently, we retrieved the *C. freundii Cf*-Emp plasmid pCf-VIM (CP110776.1) for comparison analysis, given its highest similarity to these hits (coverage: 56%; identity: 100%). (ii) For the region spanning 23,079–37,721 bp, 28 sequences (Supplementary Table S3) were matched, including 25 from *E. coli*, 1 from *C. freundii*, 1 from *K. oxytoca*, and 1 from *K. pneumoniae*. Among these, we selected the plasmid p24A20009_B_NDM from *E. coli* strain 24A20CPO009 (PV022966.1; coverage 46%, identity 99.47%), which exhibited the highest similarity, for comparison analysis. (iii) Fourteen sequences (Supplementary Table S3) mapped to the 32,773–46,714 bp region, comprising nine from *E. coli*, two from *S. enterica serovar Infantis*, and one each from *C. sakazakii*, *S. flexneri*, and *K. pneumoniae*. We selected the previously published plasmid p07D18002_A_OXA (derived from *E. coli* strain 07D18CPO002; GenBank accession PV023060.1) for comparison analysis, as it exhibited the highest similarity to the matched sequence (40% coverage; 99.97% identity). Comparison of the MDR regions of plasmids pK27-NDM5/VIM1 with those of the reference plasmids pCf-VIM, p24A20009_B_NDM, and p07D18002_A_OXA is presented in Figure 3.

### 3.6 Characteristics of plasmid pK27-KPC2

pK27-KPC2 is 134,812 bp in length with an average G + C content of 53.29% (Figure 2, Table 1). Two replicons, belonging to INCFII_pHN7A8_ and IncR types, respectively, were identified in pK27-KPC2 by PlasmidFinder. Based on these functional replicons, pK27-KPC2 was classified as an IncFII_pHN7A8_:IncR hybrid plasmid. The backbone of pK27-KPC2 is a hybrid of three regions: (i) a 30,527 bp region from pHN7A8 (GenBank no. JN232517); (ii) a 9,693 bp region from the IncR plasmid pHN84KPC (GenBank no. KY296104); and (iii) an additional 30,152 bp backbone segment derived from the IncFIIK:IncR plasmid pKPHS2 (GenBank no. CP003224), which did not harbour an intact additional replicon (Supplementary Figure S3).

The carbapenemase gene *bla*_KPC-2,_ located on plasmid pK27-KPC2, was carried by a truncated Tn*6296* element with an intact upstream region, whereas its downstream *tnpR* (derived from the internal Tn6376) was partially deleted and followed by IS*26*, yielding the structure ΔTn*1722-5′*-*ΔrepB*-*orf396*-*orf279*-*klcA*-*korC*-ΔIS*Kpn6*-*bla*_KPC-2_-IS*Kpn27*-*ΔtnpR*. A detailed schematic of this Tn*6296* variant is provided (Figure 4). Upstream of this region, an IS*26*-flanked unit, IS*26*-*bla*_SHV-12_-IS*26*, harbouring *bla*_SHV-12_, was identified; downstream, a transposon ΔTn*21*, which was largely truncated at its upstream end, was observed.

**Figure 4:**
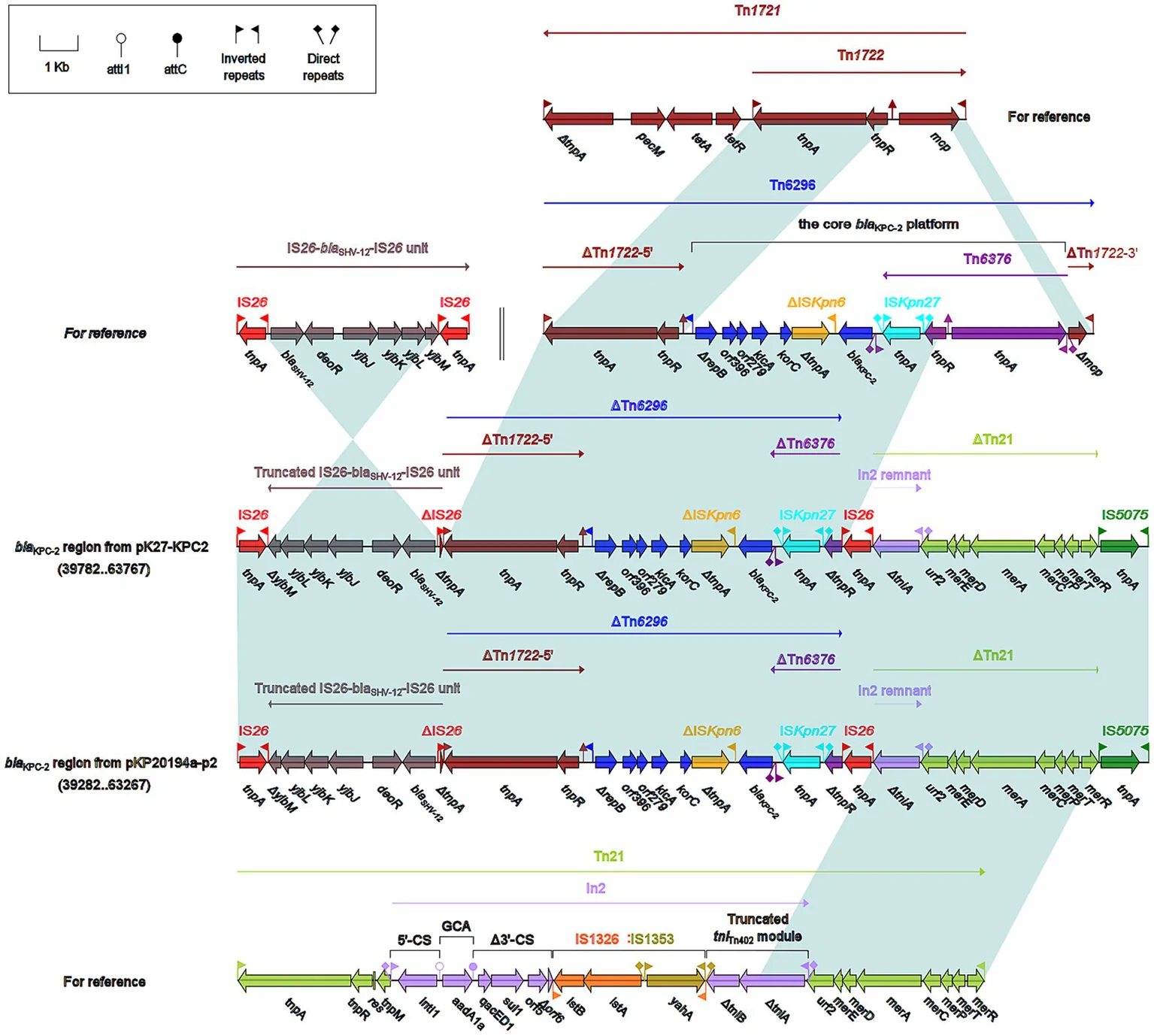
Comparison of the *bla*_KPC-2_ region between plasmid pK27-KPC2 and the reference plasmid pKP20194a-p2. Genes are denoted by arrows. Mobile elements, genes, and other features are colored based on function classification. Numbers in parentheses denote the nucleotide positions within the corresponding plasmids. Shading regions show nucleotide identity >95% calculated by BLASTn. The accession number of Tn*6296* and Tn*21* for reference are FJ628167, and AF071413, respectively.

BLASTn analysis against the NCBI database identified 85 sequences (detailed plasmid information is provided in Supplementary Table S4) with high similarity to the *bla*_KPC-2_ region of pK27-KPC2 (100% coverage, >99% identity), including 84 from *K. pneumoniae* and one from *C. freundii*. All these sequences were isolated in China, primarily in southern provinces such as Zhejiang. Given the high sequence similarity, we selected the previously published plasmid pKP20194a-p2 (Accession No. CP054782.1; 100% coverage, 99.99% identity) from *K. pneumoniae* strain KP20194a for comparison analysis. Figure 4 shows a comparison of the *bla*_KPC-2_ region between plasmid pK27-KPC2 and the reference plasmid pKP20194a-p2.

### 3.7 Characteristics of K27-chr and plasmid pK27-4

Analysis of the K27 chromosome using Kleborate (v2.3.2) with the Kaptive module revealed that it carries only the yersiniabactin locus (ybt9-ICEKp3), while lacking the genes for colibactin, aerobactin, salmochelin, and the hypermucoidy loci *rmpADC* and *rmpA2*, resulting in a virulence score of 1. The K27 strain was typed as KL64 (K64; wzi64) and O1/O2v1 (O2a). Additionally, it harboured mutations in ompK35 (OmpK35-del) and ompK36 (OmpK36-GD insertion). Furthermore, we performed a BLASTp analysis comparing the PBP3 amino acid sequence derived from the chromosome of this strain with those from *E. coli* carrying wild-type PBP3 (GenBank: WP_000642213), *E. coli* strains harbouring YRIK (GenBank: AWF10252) and YRIN (GenBank: UWB59055.1) insertions, and a *K. pneumoniae* strain without insertion (GenBank: YP_005225104). The results revealed no mutations or insertions in the PBP3 sequence of K27 strain.

Plasmid pK27-4 was also analyzed using Kleborate (v2.3.2) and Kaptive to identify virulence determinants carried on the plasmid. The results revealed that pK27-4 carries the aerobactin gene cluster (*iuc1*: *iucA*, *iucB*, *iucC*, *iucD*, and *iutA*) as well as the *rmpADC* (containing *rmpA*) and *rmpA2* genes. However, it lacks genes associated with yersiniabactin, colibactin, or salmochelin, resulting in a virulence score of 3. Furthermore, NCBI BLAST analysis identified 29 sequences highly similar to pK27-4, all exhibiting 100% query coverage and >99.97% identity. Notably, these sequences were exclusively of Chinese origin (Supplementary Table S5). Comparative analysis with the archetypal virulence plasmid pK2044 from *K. pneumoniae* NTUH-K2044 (GenBank: NC_006625) demonstrated that pK27-4 shares 92% coverage with pK2044 at 100% identity. Although both plasmids harbour the aerobactin operon (*iucABCD*-*iutA*) and the *rmpA* gene, pK27-4 is distinguished by the absence of the salmochelin locus (*iroBCDN*).

## 4 Discussion

The widespread dissemination of carbapenemase-producing Gram-negative bacteria poses a significant threat to global public health (Li et al., 2024; Paudel et al., 2024). Specifically, the rising prevalence of strains harboring multiple carbapenemase genes has rendered clinical treatment increasingly challenging (Li et al., 2026). In this study, *K. pneumoniae* strain K27, which simultaneously harboured carbapenemase genes *bla*_KPC-2_, *bla*_NDM-5_ and *bla*_VIM-1_, demonstrated resistance to first-line antimicrobial agents. Notably, strain K27 was also resistant to the novel β-lactam/β-lactamase inhibitor combinations aztreonam/avibactam and ceftazidime/avibactam. Classified as extensively drug-resistant (XDR) (Magiorakos et al., 2012), strain K27 exhibited a broad-spectrum resistance profile that significantly complicated the selection of effective clinical therapeutic options.

Bioinformatics analysis suggested that *bla*_VIM-1_ and *bla*_NDM-5_ are co-harboured on the same plasmid pK27-NDM5/VIM1. PlasmidFinder analysis revealed that the replication initiation gene *repA* of pK27-NDM5/VIM1 shares 98.32% nucleotide identity and 100% coverage with that of pRA1 (GenBank: FJ705807.1). pRA1 is the reference plasmid of the IncA group, which was historically grouped together with IncC as the “IncA/C” family (Fricke et al., 2009). However, as demonstrated by Ambrose et al., IncA and IncC plasmids are actually compatible and should be classified as distinct incompatibility groups (Ambrose et al., 2018). Accordingly, we assigned this plasmid to the IncA family in accordance with the current classification standard. The *bla*_VIM-1_ and *bla*_NDM-5_ genes were carried by a class 1 integron ΔIn916 and transposon ΔTn*125*, respectively, with the IS*CR1* located between them. In916 (GenBank accession no. KF856617) carries the gene cassette array *bla*_VIM-1_-*aacA4*-*aphA15*-*aadA1*-*catB2* in its variable region. The 3′ conserved segment (3’-CS) contains *qacEΔ1*-*sul1*, downstream of which two distinct structures have been identified: *orf5*-*ΔtniB*-*tniA*-IS*26* and *Δorf5*-*chrA*-*padR*-IS*6100*. In some isolates, the structure remained undetermined owing to contig breaks at the 5′-CS or 3′-CS boundaries (Matsumura et al., 2017; Rondinelli et al., 2026). In the present study, In916 was found to be truncated downstream of *sul1*, with IS*CR1* adjacent to the breakpoint. Additionally, compared with the original NDM-1, NDM-5 carries amino acid substitutions at positions 88 (Val → Leu) and 154 (Met → Leu) and shows increased resistance to carbapenems and broad-spectrum cephalosporins (Hornsey et al., 2011; Bahr et al., 2021). *bla*_NDM-5_ is frequently located on mobile elements such as the IS*Aba125*-based composite transposon Tn*125*. Tn*125* is a 10,099-bp transposon formed by two copies of IS*Aba125* flanking a 7,925-bp fragment (containing *bla*_NDM-1_, *ble*_MBL_, *trpF*, *dsbC*, *cutA*, *groES*, *groEL*, and IS*CR21*) and bounded by 3-bp direct repeats (target-site duplication signals for transposition). This transposon serves as a core vehicle for NDM-family genes (including *bla*_NDM-5_) and an important evolutionary platform for new variants (Chen et al., 2015; Wang et al., 2021). In contrast to the intact Tn*125*, a variant ΔTn*125* of 3,328 bp was identified in this study, extending from IS*26*-truncated IS*Aba125* to IS*CR1*-truncated *cutA*, and it harboured the carbapenemase gene *bla*_NDM-5_ instead of *bla*_NDM-1_. IS*CR1* lacks terminal inverted repeats and may transpose via a rolling-circle replication mechanism, forming composite elements. The proposed “unchecked RC transposition” mechanism, if validated, would provide a highly transferable genetic vehicle for resistance genes (Toleman et al., 2006). Previous studies suggested that IS*CR1* can locate at the 3′ conserved segment (*qacEΔ1*-*sul1*) of class 1 integrons to reconstitute “complex class 1 integrons”, and *bla*_NDM-1_ can be further inserted into the IS*CR1*-linked variable region of these complex integrons, thus forming mobile complex resistance modules (Le-Vo et al., 2019; Toleman et al., 2006). Plasmid pCF-VIM and pK27-NDM5/VIM1 are highly similar in the upstream portion of the MDR region, but they show distinct sequences after ΔIn916; the corresponding region in pCF-VIM retained a class 1 integron-derived structure, whereas that in pK27-NDM5/VIM1 was followed by IS*CR1*. Moreover, plasmid p24A20009_B_NDM shows a similar architecture in which IS*CR1* appears to serve as a bridge linking class 1 integrons ΔIn27 to the ΔTn*125* carrying *bla*_NDM-5_ (Figure 3). It is therefore postulated that IS*CR1* may have played a critical “bridge” role in the observed co-integration of *bla*_VIM-1_ and *bla*_NDM-5_, suggesting a potential mechanism for co-harbouring of these two genes on the same plasmid. The success of conjugation experiments further suggests that this mechanism may facilitate the dissemination of complex carbapenem resistance mediated by NDM-5 and VIM-1. Furthermore, conjugation experiments demonstrated that the strain K27 could transfer the plasmid pK27-NDM5/VIM1 to the recipient *E. coli* J53 Azi^R^ at a transfer frequency of (4.5 ± 1.2) × 10^−2^. This high transfer efficiency underscores the urgent need for enhanced surveillance. Acquisition of resistance plasmids confers resistance to antibiotics but often reduces bacterial fitness, typically by slowing growth rate (Nair et al., 2024). Without antibiotic selection, the plasmid-free strains generally outcompete those imposed with the metabolic burden of a plasmid (Wang Q. et al., 2023). Our *in vitro* assays showed significantly lower AUC and ODmax values, indicating that this plasmid imposes a fitness cost on *E. coli* J53Azi^R^ in the absence of antibiotic selection. Since this study only assessed the initial physiological burden via pure culture growth curves, future competition assays and serial passage experiments are needed to determine if this cost persists or if compensatory evolution promotes long-term plasmid stability.

CRKP predominantly belongs to sequence type ST258 in the United States and European countries, whereas ST11 is the major clone in China. The most important carbapenemase produced by CRKP in China is KPC (Wang et al., 2018). The *bla*_KPC-2_ gene was initially associated with transposon Tn*4401* in the United States, Colombia, and Greece; however, since 2009, China has been considered to harbour an integration of a Tn*3*-based transposon with a partial Tn*4401* structure (Shen et al., 2009). IncFII plasmids are generally low-copy-number plasmids with a narrow host range, circulating mainly among *Enterobacteriale* species, and may facilitate the spread of KPC-family genes among ST11 strains in China (Fu et al., 2019). An IncR replicon alone is capable of driving plasmid replication, but it often co-occurs with other replicons such as IncFII, IncN, IncH, and IncC (Compain et al., 2014; Fang et al., 2024). In the present study, *bla*_KPC-2_ was located on the hybrid plasmid pK27-KPC2, which is of the IncFII_pHN7A8_:IncR type. Under the experimental conditions tested in this study, pK27-KPC2 was not transferred to recipient strains via conjugation, a phenotype similar to that of the corresponding region in the comparator plasmid pKP20194a-p2. Zhang et al. hypothesized that this lack of self-transferability might be attributed to the absence of the left terminal inverted repeat of Tn*1721* (Zhang et al., 2020). Based on WGS results in this study, the backbone of pK27-KPC2 retains only remnants of conjugal transfer genes, including *slt*, *traM*, *traA*, *traL*, and *traX* (Murata et al., 2024; Ott et al., 2024). Crucially, an IS*26*-mediated insertion interrupts this transfer region, coupled with the presence of *finO*, a known fertility inhibitor (Timmis et al., 1978), which may provide a genomic basis for its experimentally observed non-transferability. However, this theoretical deficiency has not hindered the widespread existence of this resistance structure in nature. Nontransmissible plasmids such as pLVPK-like and pKPC-LK30/pHN7A8-like can be mobilized by transferable IncN3 plasmids via two mechanisms: (i) formation of hybrid plasmid fusions through replicative transposition, and (ii) simultaneous but separate transfer with the IncN3 plasmid (Wang et al., 2022). In addition, the *bla*_KPC-2_ gene has been reported to undergo IS*26*-mediated replicative transposition in clinical multidrug-resistant isolates, suggesting a possible route for its horizontal dissemination under the selective pressure of antibiotic therapy (He et al., 2015). Furthermore, recent studies indicated that the transposition of IS element from KPC-harbouring plasmid to the chromosomal capsular polysaccharide (CPS) gene cluster can disrupt CPS synthesis. This event enhanced both *in vitro* intramacrophage proliferation and *in vivo* evasion of antibiotic killing in CRKP (Xie et al., 2025). These findings on plasmid-chromosome interactions will guide our future investigations into the virulence and evolutionary dynamics of pK27-NDM5/VIM1 and pK27-KPC2 plasmids, while providing critical insights into plasmid co-integration and diversity. Consequently, this potential mechanism highlights the need for further investigation into non-conjugative evolutionary pathways.

Given the resistance of the K27 strain to aztreonam-avibactam, the chromosomal PBP3 amino acid sequence was analyzed via BLASTp against reference sequences (GenBank: WP_000642213, AWF10252, UWB59055.1, and YP_005225104) (Long et al., 2026). No mutations or insertions were identified in the PBP3 sequence. However, the K27 strain carried mutations in ompK35 (deletion) and ompK36 (GD insertion at Gly115-Asp116). These findings indicate that aztreonam-avibactam resistance is not driven by PBP3 variations, but may involve porin mutations and potentially other alternative mechanisms.

CRKP isolates harbouring the integrative and conjugative element ICEKp3, which carries the yersiniabactin-encoding gene cluster *ybt9*, have been identified across distinct clonal backgrounds (including sequence type ST11, ST340, and ST6260) in geographically separated regions (Zhu et al., 2023; Di Conza et al., 2022; Thapa et al., 2025). This provides robust genomic evidence for the global dissemination of this mobile virulence element. Similarly, K27 strain examined in this study harboured the *ybt* gene cluster together with the aerobactin synthesis genes (*iuc*) and the mucoid phenotype regulators *rmpA*/*rmpA2*. Notably, its plasmid pK27-4 shows high sequence homology to the archetypal virulence plasmid pK2044 (92% coverage, 100% identity). Collectively, these genetic features define the K27 strain as a hybrid “resistant and hypervirulent” lineage. Therefore, heightened clinical vigilance is warranted regarding the emergence of such multidrug-resistant and hypervirulent strains.

This study has several limitations. First, the analysis relied on a single clinical isolate, and no epidemiological surveillance was conducted to assess the plasmid’s prevalence within our hospital. Because the patient had several contributing causes of death, the clinical course could not be attributed specificallz to strain K27. Consequently, the present study was confined to the molecular analysis of the isolated strain. Second, we did not perform conjugation experiments with other bacterial species, nor did we evaluate plasmid stability, serial passage, or competitive growth. Addressing these gaps represents a crucial direction for future research to validate and reinforce our findings. Finally, as the fitness cost was assessed exclusively under *in vitro* conditions, it may not fully capture the complexities of clinical environments. Nevertheless, the detailed molecular characterization offers valuable baseline data for future surveillance initiatives.

## 5 Conclusion

In conclusion, *K. pneumoniae* strain K27 in this study carried three carbapenemase genes: the metallo-β-lactamase genes *bla*_NDM-5_ and *bla*_VIM-1_, both of which were located on a single conjugative IncA-type plasmid, and the serine carbapenemase gene *bla*_KPC-2_. This combination may pose considerable challenges for the selection of clinical anti-infective therapies. Moreover, the experimental demonstration of pK27-NDM5/VIM1 horizontal transfer highlights the urgent need for ongoing surveillance of such resistant strains.

## Data Availability

The plasmid sequences pK27-NDM5/VIM1 and pK27-KPC2 have been deposited in GenBank (http://www.ncbi.nlm.nih.gov/GenBank) under accession no. PZ195734 and PZ188954, respectively.

## Footnotes

1
https://www.pacb.com/

2
https://inkscape.org/en/
