# Supplementary material for: Characterization of a conjugative IncA plasmid co-harbouring blaNDM-5 and blaVIM-1 in a KPC-producing Klebsiella pneumoniae clinical isolate K27
Source: Front Microbiol. 2026 Oct 5;17:1959757. doi: 10.3389/fmicb.2026.1959757 (PMC13677901; doi:10.3389/fmicb.2026.1959757)
Supplement: Supplementary file 1 [file Supplementary_file_1.docx]

Supplementary Material-1

Figure S1. Carbapenemase production by strain K27 and its transconjugant K27-C.

Detection of carbapenemase production in strain K27 and its transconjugant K27-C using the NG‑Test® CARBA 5 assay. C indicates the quality control line; K, O, V, I, and N represent KPC, OXA-48-like, VIM, IMP, and NDM carbapenemases, respectively.

**Figure S2. S1-PFGE patterns of strain K27 and its transconjugant K27-C.**

Lane M: Salmonella enterica serovar Braenderup H9812 (PFGE marker); Lane 1: K. pneumoniae strain K27; Lane 2: K27-C conjugate.

**Figure S3. Linear comparison of plasmid pK27-NDM5/VIM1 with pRA1, and of plasmid pK27-KPC2 with pHN7A8, pKPHS2, and pHN84KPC.**.

**Linear comparison of plasmid pK27-NDM5/VIM1 with pRA1, and of plasmid pK27-KPC2 with pHN7A8, pKPHS2, and pHN84KPC.**Genes are denoted by arrows. Genes, mobile elements, and other features are colored based on the function classification. Shading denotes the regions of homology (>95% nucleotide identity). Accession numbers of pRA1, pHN7A8, pKPHS2 and pHN84KPC for reference are FJ705807.1, [JN232517](https://www.ncbi.nlm.nih.gov/nuccore/JN232517), [CP003224](https://www.ncbi.nlm.nih.gov/nuccore/CP003224) and KY296104, respectively.

**Table S1. Antimicrobial susceptibility testing profiles (MIC, mg/L)**

| **Antimicrobial class** | **Agents included** | **K27** | | **K27-C** | | ***E. coli* J53Azi^R^** | |
| --- | --- | --- | --- | --- | --- | --- | --- |
|  |  | **MIC** | **interpretation** | **MIC** | **interpretation** | **MIC** | **interpretation** |
| Penicillin | Ampicillin | ≥32 | （R） | ≥32 | （R） | 4 | （S） |
| β-lactam combination agents | Ampicillin/sulbactam | ≥32 | （R） | ≥32 | （R） | 4 | （S） |
|  | Piperacillin/tazobactam | ≥128 | （R） | 64 | （R） | ≤4 | （S） |
|  | Aztreonam/avibactam | ＞32/4 | （R） | 4/4 | （S） | 4/4 | （S） |
|  | Ceftazidime/avibactam | ＞32/4 | （R） | 16/4 | （R） | 8/4 | （S） |
| Cephems | Cefazolin | ≥64 | （R） | ≥64 | （R） | ≤4 | （S） |
|  | Ceftriaxone | ≥64 | （R） | ≥64 | （R） | ≤1 | （S） |
|  | Ceftazidime | ≥64 | （R） | ≥64 | （R） | ≤1 | （S） |
|  | Cefepime | ≥64 | （R） | ≥64 | （R） | ≤1 | （S） |
|  | Cefotetan | ≥64 | （R） | ≥32 | （R） | ≤4 | （S） |
| Monobactam | Aztreonam | ≥64 | （R） | 16 | （R） | ≤1 | （S） |
| Penems | Ertapenem | ＞16 | （R） | ＞16 | （R） | ≤0.12 | （S） |
|  | Imipenem | ＞64 | （R） | ＞64 | （R） | ≤0.12 | （S） |
|  | Meropenem | ＞64 | （R） | ＞64 | （R） | ≤0.12 | （S） |
| Amoglycosides | Amikacin | ≥64 | （R） | ≥64 | （R） | ≤2 | （S） |
|  | Tobramycin | ≥16 | （R） | ≥16 | （R） | ≤1 | （S） |
|  | Gentamicin | ≥16 | （R） | ≥16 | （R） | ≤1 | （S） |
| Quinolones | Ciprofloxacin | ≥4 | （R） | ≤0.25 | （R） | ≤0.25 | （S） |
|  | Levofloxacin | ≥8 | （R） | ≤0.25 | （R） | ≤0.25 | （S） |
| Folate pathway inhibitor | Sulfamethoxazole/Trimethoprim | ≥320 | （R） | ≥320 | （R） | ≤20 | （S） |
| Lipopeptides | polymyxinB | 0.5 | （I） | 0.5 | （I） | 0.5 | （I） |
| Tetracycles | Tigecycline | 0.5 | （S） | 0.125 | （S） | 0.125 | （S） |

The breakpoints were interpreted using the Clinical and Laboratory Standards Institute guidelines (CLSI, 2026) . FDA MIC interpretative breakpoints were used for tigecycline (Wyeth Pharmaceuticals Inc. 2016. Tygacil (tigecycline)—tigecycline injection, powder, lyophilized, for solution prescribing information. Wyeth Pharmaceuticals Inc, Philadelphia, PA). Susceptible (S); Susceptible, increased exposure (I); Resistant (R)..

**Table S2. MDR region of plasmid pK27-NDM5/VIM1**

| **Regions** | **mobile elements** | **Coordinates (Start-End)** | **Resistant genes** |
| --- | --- | --- | --- |
| **98,262–115,980 bp** | ΔTn1722 | 98,262-102,001 bp | No |
|  | ΔIn191 | 102,352-105644 bp | *dfrA14b* |
|  | truncated IS*26*-*mph(A)*-IS*6100*unit | 104,765-109,696 bp | *mph(A)* |
|  | ΔTn1722 | 109,697-111,201 bp | No |
|  | truncated IS*26*-*bla*_SHV-12_-IS*26* unit | 111,340-113,875 bp | *bla*_SHV-12_ |
|  | ΔIS*Pa17* | 113,868-115,943 bp | No |
| **115,981–129,072 bp** | ΔIn916 | 115,981-122,632 bp | *bla*_VIM-1_, *aphA15*, *aadA1b*, *aacA4'*, *catB2*, qacEΔ1 and *sul1* |
|  | IS*CR1* | 122,657-124,810 bp | No |
|  | ΔTn*125* | 124,811-128,138 bp | *bla*_NDM-5_ |
|  | IS*26* | 128,253-129,072 bp | No |
| **129,073–144,975 bp** | Tn*2*-*rmtB* element | 129,627-135,981 bp | *rmtB* and *bla*_TEM-1_ |
|  | ΔTn1721 | 136,013-137,968 bp | *tetA* |
|  | IS*CR2*-*floR* unit | 138,235-142,524 bp | *floR* |
|  | Tn6591 remnant | 140,679-143,605 bp | No |
|  | IS*26* | 144,156-144,975 bp | No |

**Table S3. plasmid information matched to the MDR region of plasmid pK27-NDM5/VIM1**

| **regions** | **No.** | **Source** | **Plasmid or Chromosome** | **Total length (bp)** | **City/Country** | **Genbank number** |
| --- | --- | --- | --- | --- | --- | --- |
|  | 1 | *Citrobacter freundii* | pCf-VIM | 151,674 | Genoa, Italy | CP110776.1 |
|  | 2 | *Citrobacter freundii* | p5370A | 167,217 | Warsaw, Poland | PQ650611.1 |
|  | 3 | *Citrobacter freundii* | p2440A | 167,526 | Warsaw, Poland | PQ650609.1 |
|  | 4 | *Citrobacter freundii* | p2980A | 163,906 | Warsaw, Poland | PQ650610.1 |
|  | 5 | *Enterobacter hormaechei* | unnamed1 | 163,083 | Salt Lake City, USA | CP132210.1 |
|  | 6 | *Enterobacter hormaechei* | unnamed1 | 225,801 | Atlanta, USA | CP190138.1 |
|  | 7 | *Enterobacter hormaechei* | p743A | 170,382 | Warsaw, Poland | OQ111274.1 |
| MDR region | 8 | *Enterobacter hormaechei* | p7753A | 161,559 | Warsaw, Poland | OQ111276.1 |
| (1–24,395 bp) | 9 | *Klebsiella oxytoca* | unnamed1 | 160,241 | Atlanta, USA | CP166430.1 |
| plasmid | 10 | *Escherichia coli* | pEco15-1 | 152,546 | Ontario, Canada | CP047711.1 |
| (98,262-122,656 bp) | 11 | *Klebsiella pneumoniae* | p7089A | 203,105 | Warsaw, Poland | PQ441979.1 |
|  | 12 | *Morganella morganii* | unnamed1 | 198,157 | Atlanta, USA | CP132322.1 |
|  | 13 | *Aeromonas sp.* | pRIVM0001_VIM-1_171012_B12 | 164,885 | Bilthoven, Netherlands | MH220284.1 |
|  | 14 | *Leclercia adecarboxylata* | Chromosome | 4,964,578 | Salt Lake City, USA | CP107262.1 |
|  | 1 | *Escherichia coli* | pNDM_2_P21_SE1_04.20 | 100,217 | London, United Kingdom | CP085076.1 |
|  | 2 | *Escherichia coli* | pYS1718-3 | 78,980 | Bern, Switzerland | CP189420.1 |
|  | 3 | *Escherichia coli* | pDC71-2 | 74,006 | Jilin, China | CP101617.1 |
|  | 4 | *Escherichia coli* | p24A20009_B_NDM | 90,753 | Manitoba, Canada | PV022966.1 |
|  | 5 | *Escherichia coli* | pMyNCGM313 DNA | 62,441 | Tokyo, Japan | LC744464.1 |
|  | 6 | *Escherichia coli* | p11A15108_A_NDM | 96,463 | Manitoba, Canada | PV023086.1 |
|  | 7 | *Escherichia coli* | pMyNCGM494_1 DNA | 82,937 | Tokyo, Japan | LC744485.1 |
|  | 8 | *Escherichia coli* | p05A18002_C_NDM | 88,692 | Manitoba,Canada | PV023028.1 |
|  | 9 | *Escherichia coli* | pEC51-NDM5 | 80,348 | Sichuan, China | OQ230786.1 |
|  | 10 | *Escherichia coli* | pSDCDK-IncFNDM5 | 80,348 | Sichuan, China | MT621569.1 |
|  | 11 | *Escherichia coli* | pMJ-T-3-88Kb | 88,906 | Jiangsu, China | CP172113.1 |
|  | 12 | *Escherichia coli* | pNDM-SDCRK18-7 | 89,325 | Sichuan, China | MN641485.1 |
| MDR region | 13 | *Escherichia coli* | pLZ135-NDM | 90,845 | Hong Kong, China | MF353156.1 |
| (23,079–37,721 bp) | 14 | *Escherichia coli* | pCUVET17-1418.2 | 96,436 | Bangkok, Thailand | CP098921.1 |
| plasmid | 15 | *Escherichia coli* | pCUVET19-1993.2 | 96,442 | Bangkok, Thailand | CP098882.1 |
| (121,340-135,982 bp) | 16 | *Escherichia coli* | p1ESCUMpO83_CORR | 92,840 | Haryana, India | CP034254.1 |
|  | 17 | *Escherichia coli* | pEC92-2_101k | 101,285 | Zhejiang, China | CP158113.1 |
|  | 18 | *Escherichia coli* | pEC1188-NDM16 | 80,215 | Zhejiang, China | MH213345.1 |
|  | 19 | *Escherichia coli* | p01B20004_C_NDM | 68,910 | Manitoba, Canada | PV022899.1 |
|  | 20 | *Escherichia coli* | pEC15-NDM-1 | 86,782 | Tokyo, Japan | LC807781.1 |
|  | 21 | *Escherichia coli* | p_dm514_NDM5 | 71,702 | Cardiff, United Kingdom | CP095625.1 |
|  | 22 | *Escherichia coli* | pA376_p2 | 80,341 | Hubei, China | CP142555.1 |
|  | 23 | *Escherichia coli* | p_b148b_NDM5 | 92,040 | Cardiff, United Kingdom | CP095579.1 |
|  | 24 | *Escherichia coli* | p_dm832c_NDM5 | 82,526 | Cardiff, United Kingdom | CP095656.1 |
|  | 25 | *Escherichia coli* | p_dm884b_NDM5 | 91,991 | Cardiff, United Kingdom | CP096181.1 |
|  | 26 | *Citrobacter freundii* | unnamed4 | 79,597 | Galway, Ireland | CP196117.1 |
|  | 27 | *Klebsiella oxytoca* | unnamed3 | 79,597 | Galway, Ireland | CP196094.1 |
|  | 28 | *Klebsiella pneumoniae* | pKC25-20651C | 98,508 | Guangdong, China | CP141990.1 |
|  | 1 | *Escherichia coli* | p07D18002_A_OXA | 138,556 | Manitoba, Canada | PV023060.1 |
|  | 2 | *Escherichia coli* | pCE780h4 | 60,848 | Brno, Czech Republic | MT859330.1 |
|  | 3 | *Escherichia coli* | pRWS531 | 118,422 | Bangkok, Thailand | CP110512.1 |
|  | 4 | *Escherichia coli* | pBL12EC-2 | 119,769 | Sichuan, China | CP079777.1 |
| MDR region | 5 | *Escherichia coli* | pqnrS | 119,424 | Sichuan, China | CP141792.1 |
| (32,773–46,714 bp) | 6 | *Escherichia coli* | pE2 | 94,190 | Hubei, China | CP086663.1 |
| plasmid | 7 | *Escherichia coli* | pZ1323CEC0001-3 | 77,526 | Iksan, South Korea | CP148509.1 |
| (131,034-144,975 bp) | 8 | *Escherichia coli* | pDX6A | 122,915 | Shandong, China | CP126346.1 |
|  | 9 | *Escherichia coli* | unnamed1 | 164,740 | Vienna, Austria | CP083884.1 |
|  | 10 | *S. Infantis strain* | pTEKIN01 | 288,036 | Altindag, Turkey | CP193652.1 |
|  | 11 | *S. nfantis strain* | pTEKIN06 | 280,709 | Altindag, Turkey | CP193643.1 |
|  | 12 | *Cronobacter sakazakii* | pCrono684-2 | 94,206 | Beijing, China | CP080596.1 |
|  | 13 | *Shigella flexneri* | unnamed1 | 99,490 | Hong Kong, China | CP058781.1 |
|  | 14 | *Klebsiella pneumoniae* | pSHX180-1 | 73,661 | Beijing, China | CP094513.1 |

**Table S4. plasmid information matched to the *bla*_KPC-2_ region of pK27-KPC2**

| **No.** | **Sources** | Plasmids | **Total length (bp)** | **City (CHINA)** | **Genbank numbers** |
| --- | --- | --- | --- | --- | --- |
| 1 | *K.pneumoniae* | p21091025_1 | 133,771 | Anhui | CP095265.1 |
| 2 | *K.pneumoniae* | pK1009_2 | 128,689 | Anhui | CP168288.1 |
| 3 | *K.pneumoniae* | p21080237_2 | 129,649 | Anhui | CP095242.1 |
| 4 | *K.pneumoniae* | unnamed3 | 133,772 | Beijing | CP167482.1 |
| 5 | *K.pneumoniae* | pK64102-KPC | 131,089 | Beijing | CP169921.1 |
| 6 | *K.pneumoniae* | pK67034-KPC | 134,477 | Beijing | CP169930.1 |
| 7 | *K.pneumoniae* | pK78927_KPC | 107,151 | Beijing | CP186939.1 |
| 8 | *K.pneumoniae* | pkp2007-KPC | 127,019 | Chongqing | CP193477.1 |
| 9 | *K.pneumoniae* | pZY1393-2 | 134,874 | Guangdong | CP076673.1 |
| 10 | *K.pneumoniae* | unnamed1 | 134,477 | Guangdong | CP101785.1 |
| 11 | *K.pneumoniae* | pB | 134,873 | Guangdong | CP090434.1 |
| 12 | *K.pneumoniae* | pNFYY328-2 | 133,774 | Guangdong | CP141647.1 |
| 13 | *K.pneumoniae* | pKP2722-2 | 134,875 | Guangdong | CP116905.1 |
| 14 | *K.pneumoniae* | pKP0079-2 | 135,440 | Guangdong | CP102838.1 |
| 15 | *K.pneumoniae* | pKP81323 | 135,468 | Guangdong | CP104315.1 |
| 16 | *K.pneumoniae* | pFAHZZU6216-2 | 133,772 | Henan | CP182921.1 |
| 17 | *K.pneumoniae* | p16ZR-187-IncFII-133-CR | 133,772 | Hong Kong | MN182746.1 |
| 18 | *K.pneumoniae* | unnamed2 | 133,114 | Hong Kong | CP087144.1 |
| 19 | *K.pneumoniae* | unnamed2 | 133,129 | Hong Kong | CP087153.1 |
| 20 | *K.pneumoniae* | pTJKP220321384-2 | 139,340 | Hubei | CP180582.1 |
| 21 | *K.pneumoniae* | pKP845_2 | 134,602 | Hubei | CP188102.1 |
| 22 | *K.pneumoniae* | pKPC3813 | 125,150 | Hubei | CP138521.1 |
| 23 | *K.pneumoniae* | unnamed2 | 134,594 | Hubei | CP107374.1 |
| 24 | *K.pneumoniae* | unnamed2 | 134,592 | Hubei | CP107385.1 |
| 25 | *K.pneumoniae* | pKP20194e-p2 | 133,772 | Hunan | CP054728.1 |
| 26 | *K.pneumoniae* | pKP20194c-p2 | 123,177 | Hunan | CP054758.1 |
| 27 | *K.pneumoniae* | pKP20194f-p2 | 133,772 | Hunan | CP054722.1 |
| 28 | *K.pneumoniae* | pKP20194a-p2 | 133,771 | Hunan | CP054782.1 |
| 29 | *K.pneumoniae* | pKP20194b2-p2 | 133,772 | Hunan | CP054764.1 |
| 30 | *K.pneumoniae* | pKP1225-P2 | 133,775 | Jiangsu | CP196488.1 |
| 31 | *K.pneumoniae* | unnamed2 | 139,321 | Jiangsu | CP172074.1 |
| 32 | *K.pneumoniae* | pNC75-5 | 134,876 | Jiangxi | CP163294.1 |
| 33 | *K.pneumoniae* | pYC2084-2 | 129,682 | Jiangxi | CP195922.1 |
| 34 | *K.pneumoniae* | pKP41-2 | 144,993 | Jiangxi | CP168668.1 |
| 35 | *K.pneumoniae* | pCRKP2-2 | 138,913 | Jiangxi | CP162936.1 |
| 36 | *K.pneumoniae* | p337-2 | 138,913 | Jiangxi | CP154492.1 |
| 37 | *K.pneumoniae* | pKPK3-2 | 132,507 | Shaanxi | CP090345.1 |
| 38 | *K.pneumoniae* | pKP0937-2 | 134,861 | Shandong | CP106762.1 |
| 39 | *K.pneumoniae* | pKP3682-2 | 149,114 | Shandong | CP141602.1 |
| 40 | *K.pneumoniae* | pKP1650-2 | 145,205 | Shandong | CP109950.1 |
| 41 | *K.pneumoniae* | p2 | 134,477 | Shanghai | CP090464.1 |
| 42 | *K.pneumoniae* | pDD02280-2 | 139,857 | Shanghai | CP087625.1 |
| 43 | *K.pneumoniae* | pDD02391-1 | 133,772 | Shanghai | CP087640.1 |
| 44 | *K.pneumoniae* | unnamed2 | 133,120 | Shanghai | CP183780.1 |
| 45 | *K.pneumoniae* | unnamed2 | 133,120 | Shanghai | CP183786.1 |
| 46 | *K.pneumoniae* | p2 | 134,670 | Shanghai | CP142693.1 |
| 47 | *K.pneumoniae* | pKPC84_130120 | 134,873 | Sichuan | CP071162.1 |
| 48 | *K.pneumoniae* | pKPC-2_SCNJ46 | 133,771 | Sichuan | PP746480.1 |
| 49 | *K.pneumoniae* | pKPC-2_SCNJ43 | 133,766 | Sichuan | PP746479.1 |
| 50 | *K.pneumoniae* | pCQPMC-KpYD1 | 132,742 | Sichuan | CP170657.1 |
| 51 | *K.pneumoniae* | pkp2020 | 287,004 | Sichuan | CP195820.1 |
| 52 | *K.pneumoniae* | pCRKP-F1-KPC | 133,859 | Sichuan | CP132044.1 |
| 53 | *K.pneumoniae* | pKPC-2_SCNJ57 | 123,420 | Sichuan | PP746485.1 |
| 54 | *K.pneumoniae* | pKPC-2_SCNJ50 | 135,683 | Sichuan | PP746481.1 |
| 55 | *K.pneumoniae* | pKPC-2_SCNJ51 | 135,810 | Sichuan | PP746482.1 |
| 56 | *K.pneumoniae* | plasB_KP36 | 149,993 | Sichuan | CP193996.1 |
| 57 | *K.pneumoniae* | pB | 134,972 | Sichuan | CP069172.1 |
| 58 | *K.pneumoniae* | unnamedB | 133,766 | Yunnan | CP146192.1 |
| 59 | *K.pneumoniae* | p3_L39 | 133,772 | Zhejiang | CP033956.1 |
| 60 | *K.pneumoniae* | pL2425-KPC-2 | 133,771 | Zhejiang | CP136269.1 |
| 61 | *K.pneumoniae* | unnamed2 | 133,770 | Zhejiang | CP031936.1 |
| 62 | *K.pneumoniae* | pKP12-KPC | 133,376 | Zhejiang | CP082767.1 |
| 63 | *K.pneumoniae* | pKP400-2 | 140,060 | Zhejiang | CP173411.1 |
| 64 | *K.pneumoniae* | pL2329-KPC-2 | 133,771 | Zhejiang | CP136845.1 |
| 65 | *K.pneumoniae* | pKP-KPC-2 | 128,928 | Zhejiang | CP084745.1 |
| 66 | *K.pneumoniae* | pJinyinhai1_KPC | 132,750 | Zhejiang | CP133403.1 |
| 67 | *K.pneumoniae* | pXH1508-2 | 133,775 | Zhejiang | CP092788.1 |
| 68 | *K.pneumoniae* | pC76-KPC | 128,928 | Zhejiang | CP080299.1 |
| 69 | *K.pneumoniae* | unnamed2 | 132,750 | Zhejiang | CP138673.1 |
| 70 | *K.pneumoniae* | pKPC-2 | 133,772 | Zhejiang | CP130265.1 |
| 71 | *K.pneumoniae* | unnamed2 | 132,750 | Zhejiang | CP138677.1 |
| 72 | *K.pneumoniae* | unnamed2 | 133,625 | Zhejiang | CP066153.1 |
| 73 | *K.pneumoniae* | pXH1507-2 | 133,775 | Zhejiang | CP092795.1 |
| 74 | *K.pneumoniae* | pKP55_2 | 133,766 | Zhejiang | CP055296.1 |
| 75 | *K.pneumoniae* | pKP36-KPC | 133,771 | Zhejiang | CP082761.1 |
| 76 | *K.pneumoniae* | pKPC-2 | 133,771 | Zhejiang | CP130259.1 |
| 77 | *K.pneumoniae* | p3 | 122,076 | Zhejiang | CP066251.1 |
| 78 | *K.pneumoniae* | p2 | 131,273 | Zhejiang | CP084707.1 |
| 79 | *K.pneumoniae* | p2 | 131,273 | Zhejiang | CP084707.1 |
| 80 | *K.pneumoniae* | pYDDB_res | 134,960 | Zhejiang | CP146353.1 |
| 81 | *K.pneumoniae* | pCRKP-35_KPC | 132,749 | Zhejiang | CP102639.1 |
| 82 | *K.pneumoniae* | p2010 | 127,576 | Zhejiang | CP171166.1 |
| 83 | *K.pneumoniae* | pICU-18 | 141,784 | Zhejiang | PX405701.1 |
| 84 | *K.pneumoniae* | pICU-3 | 141,784 | Zhejiang | PX405700.1 |
| 85 | *K.pneumoniae* | pKPC-ESBL-2 | 135,425 | Zhejiang | CP102392.1 |

**Table S5. plasmid information matched to plasmid pK27-4**

| No. | Sources | Plasmids | Total length (bp) | City (CHINA) | Genbank numbers |
| --- | --- | --- | --- | --- | --- |
| 1 | *K.pneumoniae* | pKP20194e-p1 | 195,027 | Hunan | CP054727.1 |
| 2 | *K.pneumoniae* | pKP20194c3-p1 | 195,028 | Hunan | CP054751.1 |
| 3 | *K.pneumoniae* | pKP20194c4-p1 | 195,025 | Hunan | CP054745.1 |
| 4 | *K.pneumoniae* | pKP20194c5-p1 | 195,034 | Hunan | CP054739.1 |
| 5 | *K.pneumoniae* | pKP20194f-p1 | 194,901 | Hunan | CP054721.1 |
| 6 | *K.pneumoniae* | pKP20194a2-p1 | 195,030 | Hunan | CP054775.1 |
| 7 | *K.pneumoniae* | pKP20194b2-p1 | 195,077 | Hunan | CP054763.1 |
| 8 | *K.pneumoniae* | pKP20194c-p1 | 195,024 | Hunan | CP054757.1 |
| 9 | *K.pneumoniae* | pKP20194b-p1 | 195,034 | Hunan | CP054769.1 |
| 10 | *K.pneumoniae* | pKP20194a-p1 | 195,031 | Hunan | CP054781.1 |
| 11 | *K.pneumoniae* | pKP20194d-p1 | 194,896 | Hunan | CP054733.1 |
| 12 | *K.pneumoniae* | pVIR3813 | 194,581 | Hubei | CP138524.1 |
| 13 | *K.pneumoniae* | unnamed1 | 193,826 | Hubei | CP107321.1 |
| 14 | *K.pneumoniae* | unnamed1 | 193,826 | Hubei | CP107335.1 |
| 15 | *K.pneumoniae* | unnamed1 | 193,827 | Hubei | CP107328.1 |
| 16 | *K.pneumoniae* | unnamed1 | 194,591 | Hubei | CP107384.1 |
| 17 | *K.pneumoniae* | unnamed1 | 193,835 | Hubei | CP107373.1 |
| 18 | *K.pneumoniae* | unnamed1 | 193,834 | Hubei | CP107346.1 |
| 19 | *K.pneumoniae* | pKP845_1 | 193,822 | Hubei | CP188101.1 |
| 20 | *K.pneumoniae* | p1 | 200,047 | Jiangxi | CP174256.1 |
| 21 | *K.pneumoniae* | pJX9-1 | 277,720 | Jiangxi | CP064212.1 |
| 22 | *K.pneumoniae* | pJX10-1 | 394,328 | Jiangxi | CP064259.1 |
| 23 | *K.pneumoniae* | p1 | 201,125 | Zhejiang | CP084706.1 |
| 24 | *K.pneumoniae* | pVir-1 | 193,821 | Zhejiang | CP102391.1 |
| 25 | *K.pneumoniae* | p1b1 | 200,216 | Zhejiang | CP034124.1 |
| 26 | *K.pneumoniae* | pkp2007-VIR | 195,008 | Chongqing | CP193476.1 |
| 27 | *K.pneumoniae* | pFAHZZU6216-1 | 194,981 | Henan | CP182920.1 |
| 28 | *K.pneumoniae* | pKP1225-P1 | 200,528 | Jiangsu | CP196487.1 |
| 29 | *K.pneumoniae* | unnamedA | 193,835 | Yunnan | CP146191.1 |
